# Pediatric pulmonary valve replacements: Clinical challenges and emerging technologies

**DOI:** 10.1002/btm2.10501

**Published:** 2023-03-01

**Authors:** Matthew Crago, David S. Winlaw, Syamak Farajikhah, Fariba Dehghani, Sina Naficy

**Affiliations:** ^1^ School of Chemical and Biomolecular Engineering The University of Sydney Sydney Australia; ^2^ Department of Cardiothoracic Surgery Heart Institute, Cincinnati Children's Hospital Cincinnati OH USA

**Keywords:** biofouling, pediatric, pulmonary valve replacement, somatic growth

## Abstract

Congenital heart diseases (CHDs) frequently impact the right ventricular outflow tract, resulting in a significant incidence of pulmonary valve replacement in the pediatric population. While contemporary pediatric pulmonary valve replacements (PPVRs) allow satisfactory patient survival, their biocompatibility and durability remain suboptimal and repeat operations are commonplace, especially for very young patients. This places enormous physical, financial, and psychological burdens on patients and their parents, highlighting an urgent clinical need for better PPVRs. An important reason for the clinical failure of PPVRs is biofouling, which instigates various adverse biological responses such as thrombosis and infection, promoting research into various antifouling chemistries that may find utility in PPVR materials. Another significant contributor is the inevitability of somatic growth in pediatric patients, causing structural discrepancies between the patient and PPVR, stimulating the development of various growth‐accommodating heart valve prototypes. This review offers an interdisciplinary perspective on these challenges by exploring clinical experiences, physiological understandings, and bioengineering technologies that may contribute to device development. It thus aims to provide an insight into the design requirements of next‐generation PPVRs to advance clinical outcomes and promote patient quality of life.

**Abbreviations**
CHD: congenital heart disease
ECM: extracellular matrix
EOA: effective orifice area
ePTFE: expanded polytetrafluoroethylene
FBGC: foreign body giant cell
FBR: foreign body reaction
FXII: Factor XII
HVR: heart valve replacement
PEG: poly(ethylene glycol)
PPM: prosthetic‐patient mismatch
PPVR: pediatric pulmonary valve replacement
PV: pulmonary valve
RVOT: right ventricular outflow tract
SGAHV: synthetic growth‐accommodating heart valve
SVD: structural valve deterioration
TEHV: tissue engineered heart valve
TPVR: transcatheter pulmonary valve replacement
vWF: von Willebrand's factor

## 1 INTRODUCTION

Congenital heart diseases (CHDs) are present in around 1%–2% of live births,^1^, ^2^, ^3^, ^4^ with one‐third of patients requiring hospitalization during infancy.^5^ In recent decades, diagnostic advances have improved CHD detection, while surgical advances have greatly reduced CHD‐related mortalities.^3^ Many CHDs affect the right ventricular outflow tract (RVOT) (Table 1), leading to a significant requirement for pulmonary valve (PV) replacement in the pediatric population.^12^, ^13^, ^14^, ^15^ The PV is one of four heart valves that mediate a strict unidirectional blood flow through the heart^16^, ^17^; however, the lack of ideal replacement prosthetics generates a requirement for repeat interventions throughout early life.^18^, ^19^ Prosthetics based on metals are prone to thrombosis and require lifelong anticoagulation, introducing additional burdens that persist past childhood,^20^, ^21^ while those based on biological materials exhibit underwhelming durability due to their immunogenicity and structural degradation.^22^, ^23^ Prosthetic failure is generally exacerbated in the pediatric population due to their active lifestyle and robust immune responses accelerating adverse biological responses and structural degradation.^24^, ^25^ Furthermore, the somatic growth of pediatric patients is not accommodated by current PV prosthetics, which all operate at a fixed diameter.^12^, ^14^, ^26^, ^27^ A growing mismatch between patient and prosthetic sizes gradually impairs hemodynamic performance and cardiovascular health.^24^ Taken together, these failure modes result in an unacceptably high requirement for reoperation in the pediatric population, leaving the patient and family under sustained physical, emotional, and financial hardships.^28^, ^29^, ^30^ Compared to unaffected children, those with CHD spend twice as long in hospital at 10 times the expense, and their risk of early mortality is 10‐fold.^28^ Within 10 years of RVOT intervention, pediatric patients can expect to spend more than 80 days in hospital at a cost of over $200,000 (USD, 2017), with a large portion of this time and money dedicated to reintervention.^29^ Thus, there is an urgent demand for the development of novel pediatric PV replacements (PPVRs) that may reduce the current reoperation requirements for pediatric patients with CHD. Furthermore, over 90% of CHD patients now survive into adulthood, resulting in a growing lifetime prevalence of adult congenital heart disease^9^, ^15^, ^31^; for example, the number of people aged between 50 and 69 years old living with CHD has increased by 117% since 1990.^3^ Heart valve reinterventions are the most common procedure in adult CHD,^15^ further highlighting the need for more durable prosthetics that can reduce reoperations later in life. To this end, various emerging bioengineering technologies may provide the platform necessary for the invention of better PPVRs with long‐term utility. This review first examines the functional requirements of PPVRs, the catalogue of contemporary prosthetics, and their clinical outcomes to concisely convey the nature and urgency of this issue. Then, the physiological mechanisms underlying PPVR failure is explored to facilitate a discussion of the bioengineering advances that may facilitate the innovation of next‐generation PPVRs.

**TABLE 1:** Common CHDs requiring PPVR implantation.

Disease	Description	Heart valves affected	Percentage of CHDs (%)^2^	Treatment strategy
Tetralogy of Fallot	A complex commonly consisting of a ventricular septal defect, overriding of the aorta, right ventricular outflow obstruction and right ventricular hypertrophy^1^, ^6^	Pulmonary	4.4	Surgical repair often involves pulmonary valvectomy. A typical sequela is pulmonary regurgitation, which may necessitate pulmonary valve replacement^7^
Truncus arteriosus	Lack of separation between the left ventricular outflow tract (LVOT) and RVOT, resulting in a single valved conduit that separates into the aorta and pulmonary artery^8^	Pulmonary, aortic	1.0	The pulmonary artery is separated from the main trunk, and continuity between the right ventricle and pulmonary artery is typically achieved with a valved conduit^8^
Transposition of the great arteries	Incorrect alignment of the ventricles and great arteries; right ventricle flows into the aorta while the left ventricle flows into the pulmonary artery, sometimes associated with septal defects or pulmonary stenosis^1^	Pulmonary, aortic	3.8	Surgical strategy depends on diagnosis, however, when there is severe pulmonary stenosis, reconstruction of the RVOT may be required with a valved conduit^7^
Pulmonary stenosis	Abnormal stiffening of the pulmonary valve, inhibiting opening during systole. Reported in 7% of all CHD cases^9^	Pulmonary	6.2	Balloon valvuloplasty or surgical valvotomy (for moderate–severe pulmonary stenosis)^7^
Pulmonary regurgitation following treatment for pulmonary stenosis	Pulmonary valve insufficiency commonly occurs as a sequela of balloon valvuloplasty^7^	Pulmonary	–	Valve replacement (for moderate or greater pulmonary regurgitation and right ventricular enlargement)^7^
Congenital aortic valve diseases	For example, bicuspid aortic valve^10^	Aortic, pulmonary	4.6	Aortic valve disease be treated by transplantation with the patient's own pulmonary valve in the Ross procedure, thus requiring a valve replacement in the PV position^11^

*Note*: Note that disease frequencies underrepresent the frequency of PPVR implantation as patients inevitably require multiple procedures.

## 2 PHYSIOLOGY OF THE PULMONARY VALVE

**FIGURE 1:**
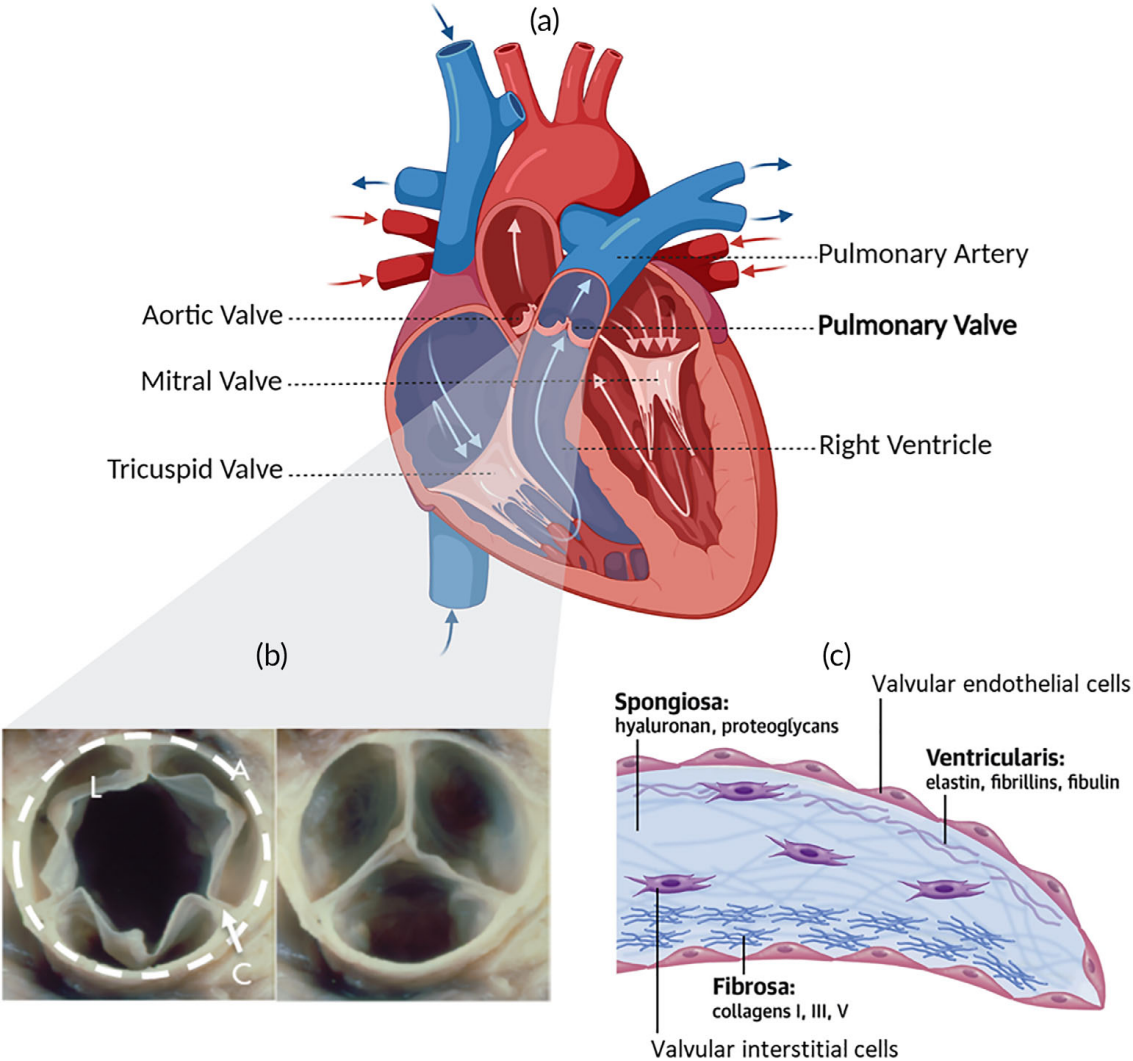
Pulmonary valve structure. (a) Unidirectional blood flow through the heart is facilitated by four heart valves (pulmonary, aortic, mitral, and tricuspid). The pulmonary valve mediates flow between the right ventricle and pulmonary artery. (b) A semilunar valve during systole (left) and diastole (right), marking the leaflet (L), annulus (A) and commissural area (C). Adapted from Schoen et al. 2018^32^; (c) Cellular and extracellular composition of a heart valve leaflet. Adapted from del Monte‐Nieto et al.^33^

The PV is an important structure in right heart function as it mediates unidirectional blood flow from the right ventricle into the pulmonary artery by preventing backflow during diastole^16^, ^32^ (Figure 1a). PV dysfunction is characteristic of many CHDs and their surgical repairs, such as in Tetralogy of Fallot,^1^, ^6^ truncus arteriosus,^8^ and transposition of the great arteries.^1^, ^34^ In health, the PV consists of three crescent‐shaped leaflets anchored to a crown‐shaped annulus, itself adjoined to the myocardium of the RVOT.^35^ Anatomically, the sinuses describe the spaces between each leaflet and the RVOT wall, the commissures describe the points along the annulus where two leaflets meet, and the interleaflet triangles describe the regions underneath each commissure^35^ (Figure 1b). At the microstructural level, the leaflet extracellular matrix (ECM) exhibits three distinct layers: the fibrosa, spongiosa and ventricularis^16^, ^32^, ^33^ (Figure 1c). The fibrosa layer is closest to the arterial surface of the leaflet and is characterized by a dense network of circumferentially aligned Type I collagen fibers,^35^, ^36^ which continues into the annulus via the commissures.^32^, ^35^ When the valve is open, the collagen occupies a corrugated configuration, which uncrimps as the valve closes.^36^ On the ventricular side of the leaflet, networks of radially aligned elastin fibers form the ventricularis layer.^36^, ^37^ In between the fibrosa and ventricularis, the spongiosa is predominated by highly hydrated proteoglycans and glycosaminoglycans.^37^, ^38^ A suite of other proteins (such as fibronectin, periostin, and vitronectin) are also present throughout the ECM to maintain mechanical integrity and mediate cellular activity.^38^ The valvular surface is lined with a circumferentially orientated monolayer of valvular endothelial cells, while its interior is populated by valvular interstitial cells.^37^, ^38^ Pathological valvular interstitial cell differentiation into an osteoblast‐like phenotype stimulates the upregulation of osteogenic genes and deposition of calcium phosphates, resulting in calcific valvular diseases.^16^, ^32^, ^38^

**FIGURE 2:**
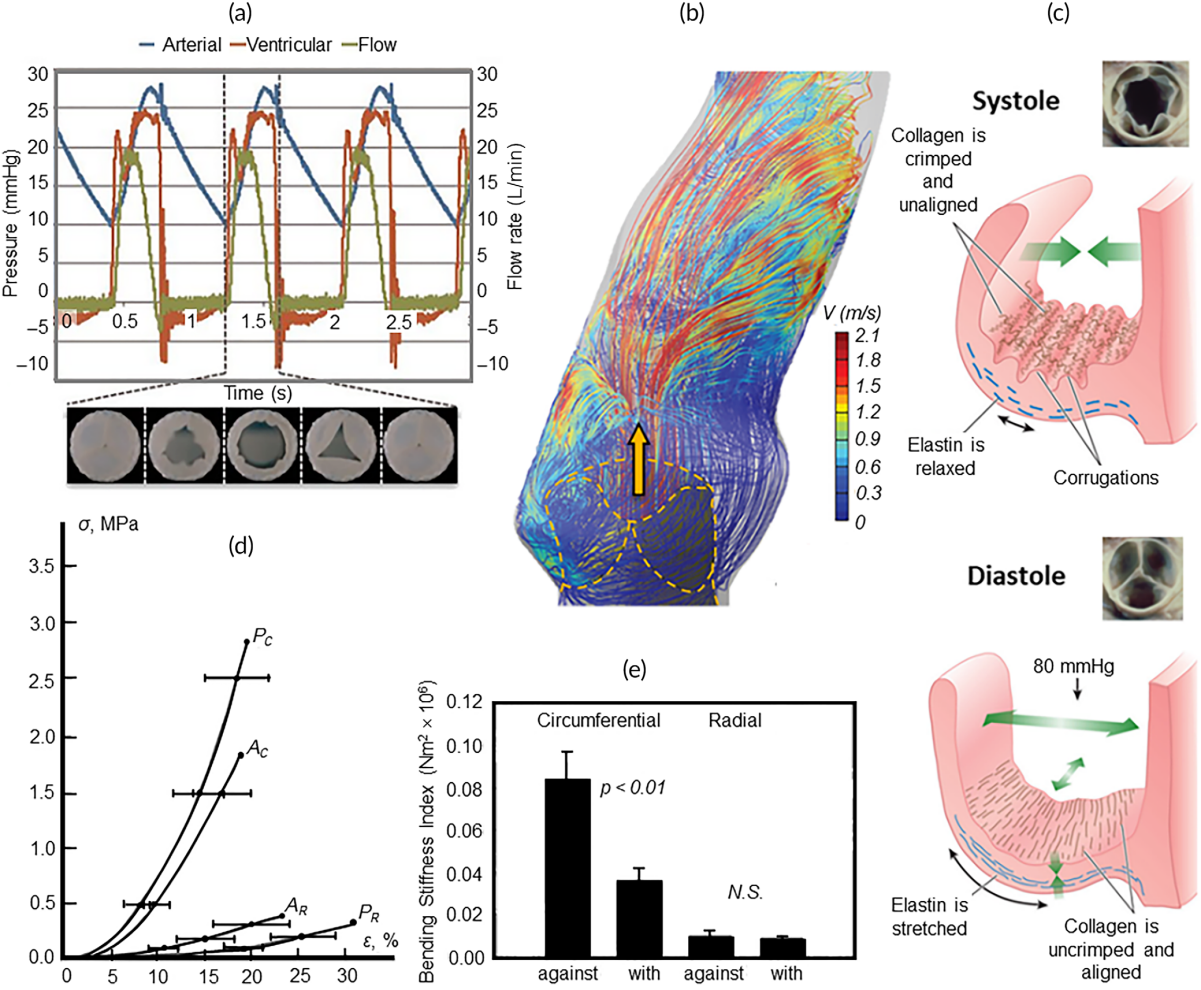
Pulmonary valve function. (a) Pressure profiles in the right ventricle and pulmonary artery, and flow profile through the PV during the cardiac cycle. Adapted from Emmert et al. 2018.^39^ (b) Flow simulation through a prosthetic aortic valve, noting the vortices formed in the sinuses. Adapted from Sotiropoulos et al. 2016.^40^ (c) Visualization of the structure–function relationship between leaflet ECM and valve opening. Adapted from Schoen et al. 2018.^32^ (d) Tensile properties of aortic (A) and pulmonary (P) valve leaflets in the circumferential (C) and radial (R) directions. Adapted from Stradins et al. 2004.^41^ (e) Anisotropic flexural properties of porcine heart valve leaflets. Adapted from Gloeckner et al. 1999.^42^

As this review aims to provide a resource for the development of next‐generation PPVRs, an understanding of their biomechanical requirements is important. In this respect, the structural and functional similarities between the PV and the aortic valve, collectively termed semilunar valves,^35^, ^37^ allow the translation of aortic‐specific biomechanical studies to this exploration of the PV. The PV opens and closes with each cardiac cycle (Figure 2a), summing to at least 3 billion cycles over an individual's lifetime.^16^, ^17^ During systole, the PV opens to eject blood into the pulmonary artery and closes during diastole to prevent backflow.^16^, ^32^ Systolic opening is induced by the contraction of the right ventricle, generating pressures around 25 mmHg to drive blood through the PV.^43^, ^44^, ^45^ Once blood has been ejected, closure is facilitated by the rapid formation of fluid vortices in the sinus regions, occurring shortly after the inertial flow along the arterial wall reverses direction^40^ (Figure 2b). At peak diastole, the pressure gradient across the PV from the arterial surface is around 6 mmHg.^43^, ^44^ Thus, heart valves are inherently passive structures, opening and closing in response to the dynamic pressures of its cardiac environment.

Within each cycle, the PV is subject to tensile, flexural and shear stresses.^37^, ^46^ Tensile stress is applied during diastole to guard backflow, wherein elastin components initially carry the load while the collagen is still corrugated (Figure 2c, top).^36^ At around 7% strain,^47^ these collagen corrugations flatten, absorb the tensile load, and transfer it into the pulmonary root^36^ (Figure 2c, bottom); ultimately, the collagen‐rich fibrosa bears the majority of the tensile load.^37^ This microstructural heterogeneity establishes a nonlinear stress–strain profile^46^ and anisotropic tensile properties; leaflets tend to be stiffer and stronger in the circumferential direction but more extensible in the radial direction^41^, ^48^ (Figure 2d). Flexural stresses predominate the stress cycle, as flexure is the principal deformation mode in heart valves.^36^ Based on the assumptions of Euler–Bernoulli simple beam theory, leaflets bent around the circumferential plane are twice as stiff when bending against their natural curvature as compared to bending with their natural curvature.^42^, ^49^ Conversely, no differences in flexural stiffness are observed when bending around the radial plane^42^ (Figure 2e). Functionally, this facilitates valve opening but prevents inversion during closing.^42^, ^49^ This is permitted by the heterogeneous microstructure of the leaflets, with the stiffer fibrosa on the concave surface and the extensible ventricularis on the convex surface.^36^, ^37^ Finally, blood flowing through the open valve imparts shear stresses on the leaflets, which are largest during early systole; for example, under normal adult resting conditions (70 bpm and 5 L/min cardiac output), the aortic valve typically experiences maximum stresses of around 7 Pa on the inflow leaflet surface^50^, ^51^ and 2 Pa on the outflow surface.^52^

## 3 CURRENT PEDIATRIC PULMONARY VALVE REPLACEMENTS

**FIGURE 3:**
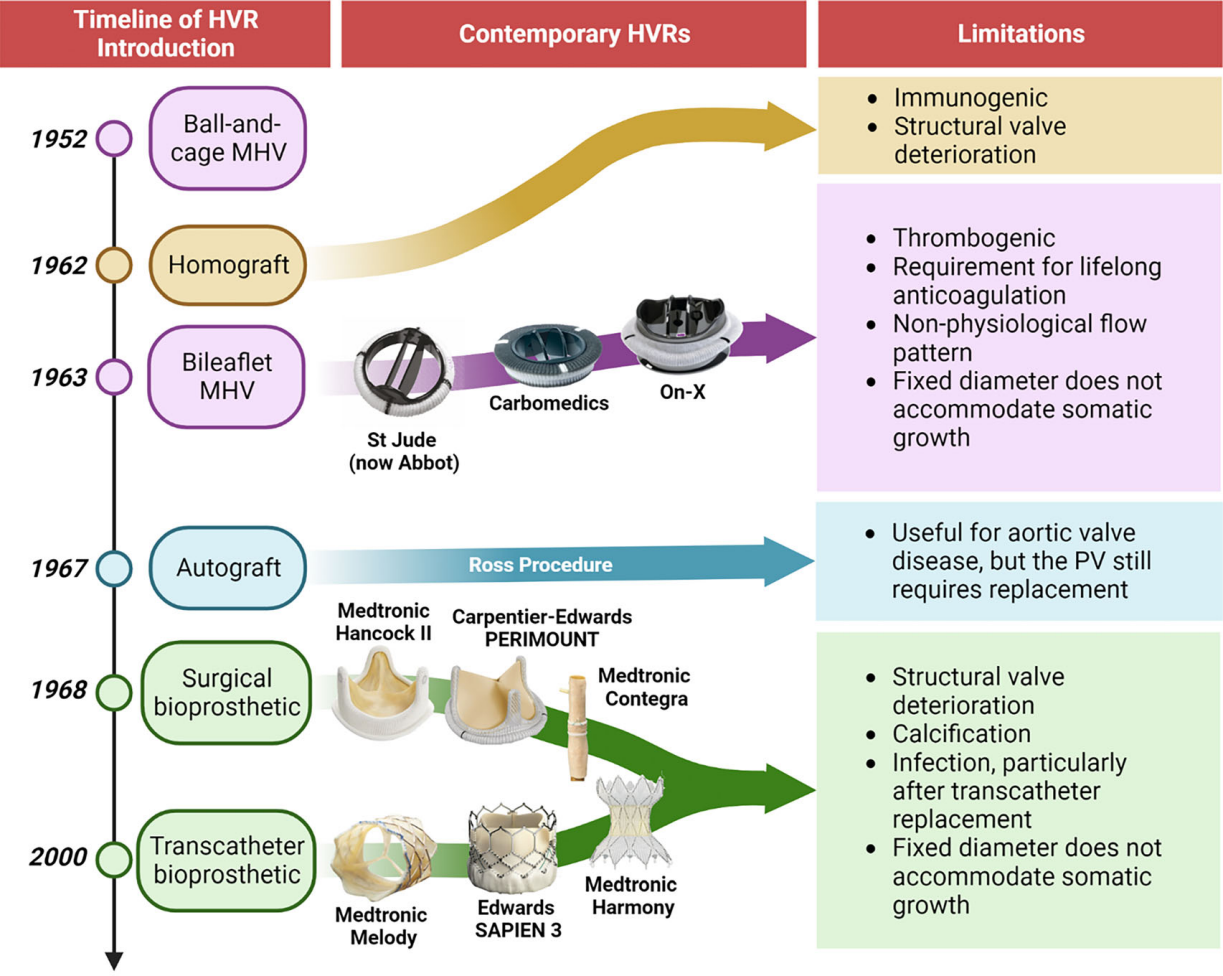
Introduction timeline of HVRs with application in the pediatric population. The first ball‐and‐cage mechanical valve was introduced in 1952,^55^ with the first generation of bileaflet mechanical valves introduced in 1963.^56^ The first homograft was implanted in 1962,^57^ the first use of an autograft in the Ross procedure was reported in 1967.^58^ The first chemically‐treated bioprosthesis was described in 1968^59^ and the first transcatheter bioprosthesis was successfully deployed in 2000.^60^

The first‐line treatment for congenitally diseased PVs is almost always repair in order to preserve the native tissue.^12^, ^14^ For example, pulmonary stenosis may be treated with balloon pulmonary valvuloplasty, wherein the expansion of a transcatheter balloon forces the stenotic leaflets open.^9^, ^53^, ^54^ However, PV replacement is often inevitable,^12^, ^14^ such as when valvuloplasty or valvectomy eventuates in significant pulmonary regurgitation.^9^ A range of heart valve replacements (HVRs) have been introduced since the 1950s, including mechanical valves, bioprosthetic valves homografts and autografts (Figure 3). In recent years, an emerging family of polymeric valves have been explored; however, none have yet achieved clinical and commercial translation. No ideal PPVR is currently available, with all contemporary options exhibiting limitations that preclude extended durability, thus requiring patients to undergo multiple surgeries.

### 3.1 Mechanical valves

Mechanical valves are constructed from rigid materials such as steel, titanium alloys, and pyrolytic carbon.^56^ In the pediatric population, the use of mechanical valves is restricted to implantation in the systemic circulation with adequate outcomes.^61^, ^62^ Importantly, mechanical valves are rarely used in the pulmonary circulation due to incompatibilities with right ventricular pressures and an exacerbated requirement for anticoagulation. In general, novel PPVRs would likely be utilized in place of bioprosthetic valves; however, if their performance and durability are suitable then they could also be considered in cases where mechanical valves would be selected, warranting a brief discussion on the limitations of mechanical valves. Thrombosis is a persistent concern for mechanical valves, attributed to their artificial metallic construction that promotes biofouling^20^, ^24^, ^63^ and non‐physiological flow profile that induces cavitation.^64^ Prevention of thrombosis is managed by pharmaceutical treatment with vitamin K antagonists^20^ and more recently a range of novel oral anticoagulants such as dabigatran, rivaroxaban, apixaban and edoxaban.^65^ However, lifelong anticoagulation predisposes bleeding risks,^24^ requires patients to adhere to a strict pharmaceutical routine, and necessitates regular blood tests to monitor treatment effect and to adjust dosage.^20^ Furthermore, all mechanical valves function at a fixed diameter, thus precluding the accommodation of somatic growth that occurs during early life. While some surgical strategies involve deliberately dilating the native annulus to implant an oversized prosthetic, this process involves additional incisions that can inadvertently damage ventricular function and the conduction pathway.^12^

### 3.2 Bioprosthetic valves

Bioprosthetic valves are constructed from biological tissues, often in combination with metallic or polymeric supports, and demonstrate more physiological flow profiles and reduced susceptibility to thrombosis than mechanical valves. Bioprosthetics are commercially available for surgical or transcatheter delivery,^66^, ^67^ and are treated with glutaraldehyde to preserve structural integrity and reduce immunogenicity.^24^ Various bioprosthetics have historically been used as PV replacements, including the Hancock II (a stented porcine aortic valve),^66^ the Carpentier‐Edwards PERIMOUNT (a stented bovine pericardial valve)^66^, ^68^ and the Freestyle (a porcine aortic root).^69^ In 1999, the bovine jugular vein conduit (BJVC) emerged as a promising graft for RVOT reconstruction due to the valve being naturally integrated within the conduit.^70^ A BJVC for surgical delivery was commercialized as the Contegra valve (Medtronic, USA), with utility in RVOT diameters between 12 and 22 mm.^69^, ^70^ The advent of transcatheter PV replacement (TPVR) in 2000^60^ introduced a non‐invasive alternative to surgery,^54^, ^67^, ^71^ while achieving the same hemodynamic performance.^72^ Today, TPVR is preferred to surgical methods^54^ and many transcatheter PPVRs are commercially available,^54^, ^67^ although surgical strategies remain common in younger patients (<30 kg) due to size constraints for TPVR vascular access. The seminal TPVR procedure commercially translated into the Melody valve (Medtronic, USA), which consists of a BJVC in a balloon‐expandable platinum‐iridium stent^73^, ^74^ with utility in RVOT diameters between 16 and 24 mm.^71^ The SAPIEN family of transcatheter PPVRs, consisting of a trileaflet valve constructed from bovine pericardium in a chromium stent, was then introduced in 2006^75^, ^76^ for RVOT diameters up to 29 mm,^71^ with clinical trials underway for its third generation (SAPIEN 3, NCT02744677). TPVR in diameters larger than 29 mm remained a limitation for both Melody and SAPIEN devices,^23^, ^77^ catalyzing the development of newer TPVR generations, such as the Harmony valve (Medtronic, USA),^76^, ^78^, ^79^ Alterra Adaptive Prestent (Edwards Lifesciences, USA),^80^, ^81^ and Venus P (Venus MedTech, China).^82^, ^83^ All these devices utilize an hourglass‐shaped self‐expandable nitinol stent, with the Harmony and Venus P devices incorporating valves made from porcine pericardial tissues^25^, ^82^ and the Alterra Adaptive Prestent providing a landing site for the SAPIEN valve.^81^

The longevity of bioprosthetic valves is limited by their susceptibility to structural valve deterioration (SVD),^22^ which describes the progressive degradation of the bioprosthetic tissue, resulting in hydrodynamic dysfunction.^23^, ^77^ Calcification is typical of SVD^84^ and leads to leaflet stiffening,^25^, ^85^ while various other hallmarks include immune responses and the foreign body reaction (FBR),^23^, ^83^, ^85^ mechanical degradation,^23^, ^82^, ^86^ and the deposition of advanced glycation end products.^87^ As the nonviable bioprosthetic tissue lacks repair capabilities, these myriad factors accumulate toward significant structural failure.^77^, ^88^ The exacerbation of SVD in the younger population is generally attributed to the more active lifestyle of this demographic accelerating the wear on the prosthetic. It may also emerge from the more vigorous immune system of younger patients,^23^, ^24^, ^25^ and the lack of growth potential in contemporary fixed‐diameter bioprostheses.^14^ While the Melody valve can be slightly over‐dilated to correct increasing transvalvular pressures,^89^, ^90^, ^91^ there is no evidence for any long‐term capacity in accommodating growth. Bioprostheses may also fail due to infective endocarditis or thrombosis.^22^ Infective endocarditis is an infection of the blood facing surfaces around implanted HVRs,^92^, ^93^ with reintervention typically required due to its severity.^71^, ^72^, ^94^, ^95^ It is predominantly a concern after transcatheter procedures, with an incidence between 7.5% and 17% after TPVR.^96^, ^97^ Endocarditis occurs more in BJVCs than in homografts, and the transcatheter Melody valve exhibits significantly greater susceptibility than the surgical Contegra valve.^98^ Finally, thrombosis is relatively rare in bioprosthetics, and it is remarkedly less concerning than in mechanical valves with events often limited to the first 3 months after implantation as the device undergoes endothelialization.^99^, ^100^ In fact, the mortality associated with bioprosthetic valve reoperation is significantly less than that associated with mechanical valve bleeding events.^100^

### 3.3 Homografts

Homografts are transplanted from human donors, demonstrating considerably greater durability and better freedom from reoperation than bioprosthetics.^101^ However, they suffer SVD at unpredictable rates and valve competence in the medium term is variable. Reoperation to replace the homograft is frequent and younger age at implantation continues to be a risk factor for reoperation,^101^ again likely due to somatic outgrowth and immune responses.^13^ Some groups have suggested that decellularizing the homograft may improve longevity by removing immunogenic material.^102^ For example, decellularized homografts implanted in the pulmonary position of adolescents and young adults have reported slightly better durability than standard homografts and bovine jugular veins.^103^ However, there is no clinical consensus on the importance of decellularization, with many studies demonstrating no benefit to homograft longevity.^104^, ^105^ Furthermore, immunogenicity is not entirely eliminated by decellularization, and implants may still experience immune responses against residual cellular components or remnants of detergents used in the decellularization process.^106^ Nonetheless, homografts are an excellent graft in comparison to bioproshtetics,^19^, ^101^ although their application remains imperfect due to their limited availability and inevitable SVD.

### 3.4 Polymeric valves

The persistent inadequacies of available HVRs have catalyzed the development of polymeric valves that utilize synthetic polymers to combine the durability of mechanical valves with the thromboresistance and hydrodynamic profile of bioprosthetic valves. The first polymeric valve was reported in 1958^107^ and the first in‐human implantation occurred in 1960,^108^ and despite decades of research, polymeric valves have yet to undergo clinical translation. Various polymers have been explored, including expanded polytetrafluoroethylene (ePTFE),^109^, ^110^ poly(styrene‐*b*‐isobutylene‐*b*‐styrene),^111^, ^112^ and polyurethane.^113^, ^114^, ^115^, ^116^ The closest device to clinical translation is a recent siloxane‐based polyurethane surgical valve developed by Foldax (https://foldax.com/).^113^ In a small population (*n* = 15) trial, quality of life was improved for one‐third of patients 1 year after aortic valve repalcement.^117^ Further clinical trials in the aortic (NCT03851068) and mitral (NCT04717570) positions are underway with expected completion in 2026 and 2025, respectively. Moreover, a transcatheter variation is in development, with ovine trials ongoing and first‐in‐man trials expected to progress in early 2023.^118^ The utility of these polymeric valves in the pediatric population is yet to be reported.

## 4 REOPERATION REQUIREMENTS

No commercially available PPVRs are ideal and RVOT reconstruction will often require repeat operations later in life to replace dysfunctional prosthetics.^18^, ^19^ Importantly, freedom from RVOT reoperation varies depending on the clinical context, influenced by factors such as CHD etiology, age at implantation and PPVR type, as well as institutional and patient preferences. Thus, this section does not strive to provide a comprehensive analysis of PPVR reoperation rates, but rather to outline the suboptimal clinical outcomes in typical scenarios, and to explore some trends established by various clinical factors.

A recent systematic review identified the primary indications for intervention due to right heart CHDs as Tetralogy of Fallot and truncus arteriosus, and left heart CHDs commonly required right heart intervention due to the use of the PV as an autograft in the Ross procedure.^19^ PPVR implantation for truncus arteriosus tended to occur earlier in life (median age of 2.5 months), with a 10‐year freedom from reoperation of around 25%.^19^ Tetralogy of Fallot tended to require valve implantation later in life (median age of 25 years) when earlier repair procedures resulted in pulmonary regurgitation, with a 10‐year freedom from reoperation of 80%.^19^ It should be noted that these statistics were not stratified by PPVR type, although homografts were more common in truncus arteriosus, while bioprosthetics were more common in Tetralogy of Fallot.^19^ When comparing outcomes within age groups, homografts tend to exhibit superior freedom from reoperation and endocarditis than bioprosthetics^19^, ^98^, ^119^; however, younger patient age at implantation (relating to smaller PPVR diameter) has been correlated to reduced freedom from reoperation.^18^, ^19^, ^97^ For example, McElhinney et al. reported that 10‐year freedom from reoperation after TPVR for repaired Tetralogy of Fallot was around 80% in young and middle‐aged adults, but only around 60% in adolescent patients.^120^ In a younger population, Saxena et al. reported that young children receiving either BJVCs or homografts for various aetiologies (mostly truncus arteriosus) had a 10‐year freedom from reoperation of 33%, which reduced to 25% in the neonatal population.^18^

Thus, subpar reoperation requirements after RVOT reconstruction with contemporary PPVRs necessitate the development of next‐generation PPVRs with superior longevity. Two key challenges for such devices may be distilled from the preceding discussions: first, the PPVR must resist adverse biological responses to mitigate structural degradation and systemic hazards, and second, the device should ideally be able to accommodate some anatomical growth to prolong performance in growing patients. To this end, the next part of this review focuses on exploring the underlying mechanisms for these failure modes to facilitate a discussion about emerging bioengineering trends that may contribute to the development of next‐generation PPVRs.

## 5 BIOFOULING

**FIGURE 4:**
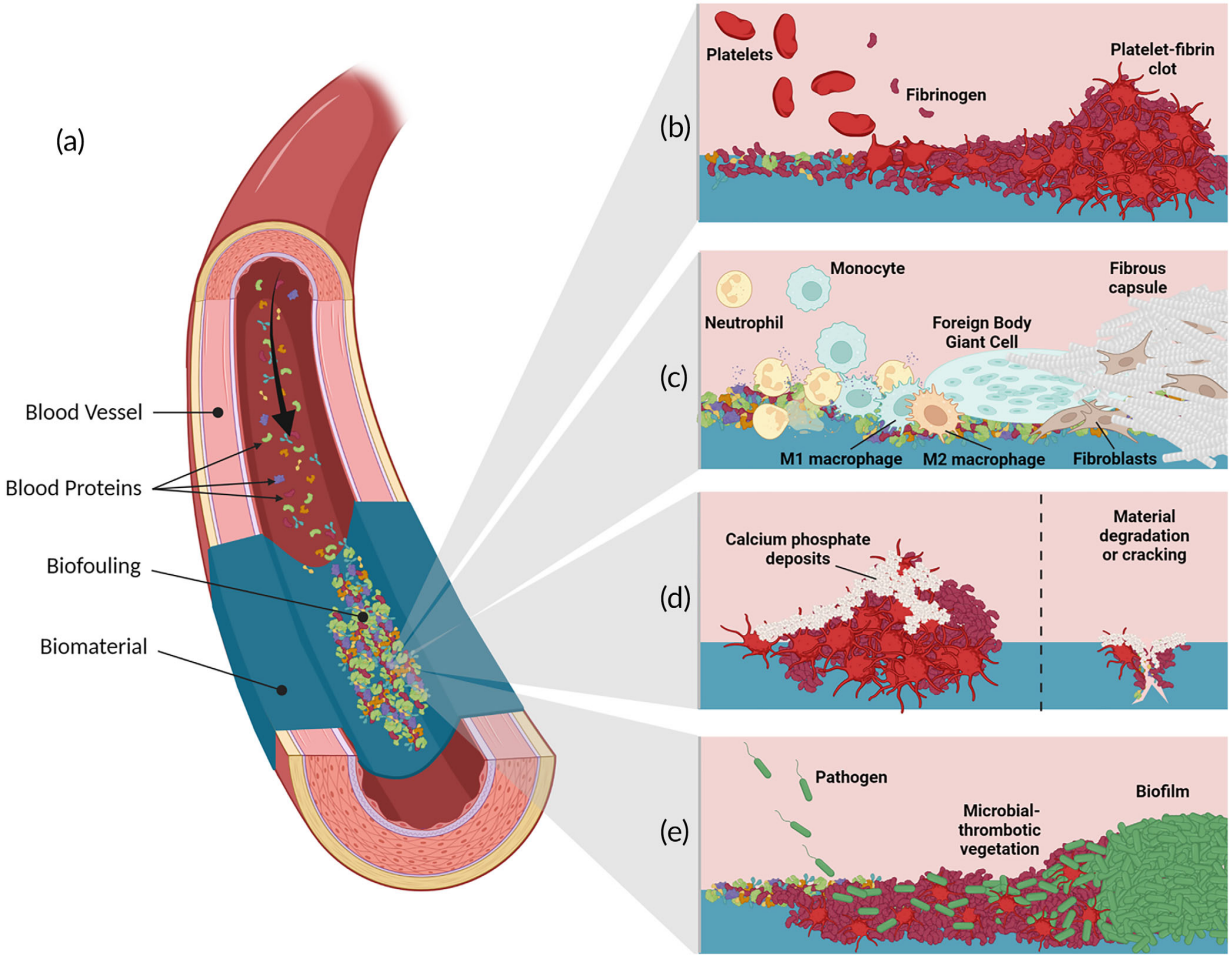
Consequences of biofouling. (a) The nonspecific adsorption of blood proteins occurs within seconds of biomaterial exposure, inducing various adverse biological events including (b) thrombosis, (c) immune responses and FBR, (d) calcification, and (e) infection. Scale and relative sizes between components are not realistic.

The nonspecific adsorption of blood proteins onto foreign materials, herein termed biofouling, occurs within seconds of exposure to blood^121^ (Figure 4a). This is thought to be associated with a suite of undesirable biological pathways that can preface HVR failure and reoperation, including thrombosis, immune responses and the FBR, calcification, and infection.

### 5.1 Consequences of biofouling

#### 5.1.1 Thrombosis

Thrombosis is a universal challenge for all blood‐contacting medical devices due to its notorious role in device failure, vessel occlusion, and systemic complications.^63^ Mechanical valves are especially susceptible to thrombosis and bioprosthetic valves have a low risk shortly after implantation, while prototype polymeric valves commonly demonstrate thrombus formation in in vivo tests. Biofouling with proteins such as fibrinogen, von Willebrand's factor (vWF) and Factor XII (FXII) is particularly important in thrombus formation^121^ (Figure 4b). For negatively charged biomaterial surfaces, the adsorption and autoactivation of FXII triggers the contact activation system, a series of proteolytic reactions resulting in thrombin generation.^63^, ^122^ FXII autoactivation is accelerated by high molecular weight kininogen,^122^ and FXII/ high molecular weight kininogen complexes are mediated by the adsorption of the complement C1q protein at the biomaterial surface.^123^, ^124^ The contact activation system culminates in the conversion of adsorbed fibrinogen to fibrin, catalyzed by thrombin, and the crosslinking of fibrin monomers, catalyzed by Factor XIIIa.^63^ Meanwhile, platelets adhere and activate to the biomaterial via adsorbed fibrinogen under low shear conditions, and via adsorbed vWF under high shear conditions, ultimately developing into thrombotic clots.^121^ Positively‐charged and hydrophobic surfaces also induce the contact activation system, albeit at a much slower rate.^121^ The mechanisms underlying thrombosis on these surfaces are not thoroughly understood, however, may be related to their tendency to denature adsorbed proteins.^121^ There is also evidence suggesting that the contact activation system may be triggered via the increased generation of neutrophil extracellular traps around hydrophobic surfaces, as these biological scaffolds are known to directly promote both FXII activation and platelet interactions.^125^ Thus, thrombosis on various biomaterial surfaces may be inhibited by avoiding the adsorption of blood proteins such as fibrinogen, vWF, FXII and C1q.

#### 5.1.2 Immune responses and the foreign body response

The FBR consists of immune‐mediated inflammatory and fibrotic pathways stimulated by the implantation of foreign biomaterials.^126^, ^127^ The first step in the FBR is the adsorption of blood proteins onto the material surface^121^, ^126^, ^128^, ^129^ (Figure 4c). The adsorption of complement proteins recruit leukocytes to the implant site to promote early inflammatory responses,^63^, ^121^, ^127^ and crosstalk between complement and coagulation components augment both inflammatory and thrombotic responses.^63^, ^130^ Acute inflammation occurs within hours of implantation, wherein neutrophils infiltrate the site to phagocytose the foreign material.^127^ Circulating monocytes are simultaneously recruited and differentiate into proinflammatory M1 macrophages,^131^, ^132^ becoming the dominant immune cell within days to mediate chronic inflammation.^126^, ^127^ In bioprosthetics, this contributes to SVD as structural proteins are degraded through proteolytic enzymes or reactive oxygen species.^23^ Where biomaterials resist degradation, such as those ideal for polymeric valves, persistent inflammation stimulates macrophages to fuse into foreign body giant cells (FBGCs) to augment phagocytic performance while avoiding apoptosis.^126^, ^131^ Importantly, FBGC formation is dependent on the extent of early fibrinogen adsorption and fibrin polymerization.^128^ Alongside FBGC formation, anti‐inflammatory M2 macrophages differentiate and stimulate myofibroblasts to deposit a collagenous matrix around the implant.^126^, ^127^ This can surround the material with a fibrotic capsule,^126^, ^131^ impairing the mechanical utility of the implanted device and isolating it from the host.^126^, ^127^ This fibrous growth may stiffen valve leaflets, and thus degrade the mechanical performance of HVRs by limiting leaflet movement.^23^ Thus, the inhibition of complement adsorption and those proteins associated with thrombosis might aid in mitigating immune responses and the FBR.

#### 5.1.3 Calcification

Calcification is a common failure mode in native heart valve tissue,^32^ bioprosthetics and many polymeric valve prototypes^116^, ^133^, ^134^, ^135^; however, its mechanism differs depending on the material (Figure 4d). In bioprosthetic valves, calcification occurs due to abnormally high calcium ion (Ca^2+^) levels in the intracellular space of devitalized tissues, enabled by the glutaraldehyde crosslinking process that damages the cell walls and deactivates cellular Ca^2+^ efflux channels.^23^, ^25^, ^82^ Interactions between intracellular Ca^2+^ and phosphorus‐rich cellular compounds, such as the cell membrane or nucleic acids, then form calcium phosphate crystals.^23^, ^25^, ^82^ Additionally, residual free aldehydes from glutaraldehyde crosslinking can react with circulating Ca^2+^ to induce calcification.^23^, ^25^, ^82^ On polymeric valves, the mechanism of calcification is less clear; however, it has been associated with thrombosis,^134^ mechanical stress and failure, and regions of low flow.^133^, ^134^, ^135^, ^136^ To this end, calcification on polymeric materials may be closely linked to thrombus formation, suggesting that inhibiting thrombus initiation may indirectly mitigate calcification.

#### 5.1.4 Infection

Infective endocarditis is a common concern after TPVR,^71^, ^72^, ^94^, ^95^ with typical causative agents including *Staphylococcus aureus*, viridians‐group *Streptococcus* subspecies, and coagulase‐negative *Staphylococci* subspecies.^97^ Early stages involve the association of microbes from transient bacteremia with thrombus at the valve site, eventually forming a microbial‐thrombotic vegetation^137^ (Figure 4e). Moreover, current models implicate fibrinogen, vWF and platelets as key intermediaries for bacterial adhesion to the endocardial surface.^138^, ^139^, ^140^ For example, *S. aureus* and *Staphylococcus lugdunensis* directly bind to vWF to overcome the high shear stresses of the valve environment.^139^, ^140^ Similarly, *S. aureus* also depends on platelets and fibrinogen^138^, ^140^; in fact, *S. aureus* adhesion has been reported to reduce threefold after medication with aspirin and ticagrelor.^138^ Infection commonly results in biofilm formation, which increases microbial tolerance to host immunity and antimicrobial treatment.^141^ Thus, infective endocarditis is facilitated by the biofouling of thrombosis‐inducing proteins such as fibrinogen and vWF, suggesting that infection may be mitigated by resisting biofouling.

### 5.2 Surface modifications for antifouling biomaterials

Thrombosis, the FBR, and infection directly depend on biofouling, while calcification is indirectly dependent via its association with thrombosis. This suggests that developing biomaterials with resistance to biofouling might mitigate these downstream consequences, allowing for the fabrication of PPVRs with long‐term resistance to biodegradation and dysfunction. Many polymeric biomaterials used in biomedical settings are chemically inert to reduce biological responses.^142^, ^143^ Out of the various polymers explored for polymeric valves, polyurethanes are most promising due to their biomimetic mechanical properties that combine strength and elasticity,^115^, ^144^ with those based on siloxane groups exhibiting the greatest resistance to biofouling, compared to those based on ester, ether or carbonate groups.^144^, ^145^, ^146^, ^147^ While polyurethanes are more resistant to biofouling than materials such as poly(ethylene terephthalate)^148^ and polytetrafluoroethylene,^149^ they are inferior to poly(styrene‐*b*‐isobutylene‐*b*‐styrene)^150^, ^151^ and ePTFE.^148^ Nonetheless, all synthetic biomaterials remain prone to biofouling,^63^, ^121^ necessitating additional strategies to diminish this susceptibility. A predominant strategy involves modifying a polymer's surface chemistry to improve resistance to biofouling at the biomaterial‐blood interface without compromising the mechanical utility of the bulk polymer.^152^ Strategies for surface modifications (Table 2) are classified as either bioactive, which actively counteract biological processes or support material repair, or passive, which aim to eliminate any interactions between the blood and bulk material.^152^

**TABLE 2:** Antifouling surface modifications with potential application in next‐generation PPVRs.

Surface modification	Class	Mechanism	Effect on biofouling	Effect on downstream events	Limitations
Heparin	Bioactive	Actively inhibits contact activation system proteins by augmenting antithrombin function^153^	None. Biofouling may actually hinder heparin mechanism^152^	Reduces thrombus formation^153^	Lack of long‐term stability.^152^ Inactive heparin may be thrombotic.^153^ May trigger heparin‐induced thrombocytopenia^154^
Tethered liquid perfluorocarbons	Passive	Prevents biomolecule adhesion with a low‐friction liquid surface, consisting of a liquid perfluorocarbon layer associated with a network of molecular tethered perfluorocarbons^155^, ^156^	Reduces fibrinogen adsorption^155^	Reduces platelet adhesion^155^ and thrombus formation.^155^ Prevents microbial adhesion and biofilm formation^155^, ^157^	Currently no evidence for long‐term stability in a cardiac environment
Poly(ethylene glycol)	Passive	Generates a hydration layer via hydrogen bonding and an energetic layer due to water displacement and PEG compression being thermodynamically unfavorable^158^, ^159^	Reduces fibrinogen adsorption^160^	Extends circulation time of modified nanoparticles in the bloodstream.^158^ Mitigates platelet adhesion and microbial adhesion^161^	Hydration layer remains susceptible to biofouling^162^ PEG oxidatively degraded in vivo^158^ Induces anti‐PEG antibodies^158^
Zwitterions	Passive	Generates a physical hydration layer via ionic bonding and an energetic barrier due to water displacement being thermodynamically unfavorable^162^, ^163^	Reduces fibrinogen adsorption^164^, ^165^, ^166^, ^167^, ^168^, ^169^, ^170^, ^171^, ^172^	Reduces platelet adhesion^164^, ^168^, ^173^, ^174^, ^175^, ^176^, ^177^ and thrombus formation.^168^, ^170^, ^171^, ^173^, ^176^, ^178^ Reduces antibody responses,^179^, ^180^ immune cell recruitment^164^, ^169^, ^175^, ^176^ and inflammation.^180^ Reduces fibroblast adhesion,^165^, ^166^, ^171^, ^174^ tissue formation^164^, ^169^ and fibrotic capsule formation.^164^ Reduces calcification^168^, ^175^, ^181^ and SVD^168^, ^181^ in bioprosthetics. Prevents adhesion of bacteria,^170^, ^171^, ^174^, ^176^, ^177^, ^182^, ^183^ viruses^182^ and fungi^171^ and limits biofilm formation^177^, ^182^, ^183^	Acidic pHs decrease surface hydration by carboxybetaine‐based zwitterions.^184^, ^185^ High ionic concentrations in solution hinder hydration by screening the zwitterionic charges.^184^ The opposite ions of sulfobetaine‐based zwitterions may self‐associate.^186^ Currently no evidence for long‐term stability in a cardiac environment

#### 5.2.1 Bioactive surface modifications

The only commercially available bioactive modification is heparin,^152^ commonly grafted using the CARMEDA method.^153^ Heparin augments the function of antithrombin, which inhibits various clotting factors in the contact activation system, such as thrombin and activated FXII.^153^ CARMEDA heparin modifications have been used to extend the lifespan of various blood‐contacting devices, including extracorporeal membrane oxygenation circuits, ventricular assist devices, coronary stents and vascular grafts.^153^ However, heparin relies on a particular five‐sugar sequence that is only present in one‐third of molecules in a commercial heparin sample, and inactive molecules may induce thrombosis due to its strong negative charge.^153^ Heparin may also be enzymatically degraded or shielded by adsorbed proteins, limiting heparin coatings to short‐term applications.^152^ Furthermore, a rare but serious side effect is heparin‐induced thrombocytopenia, an immune response to heparin complexes that causes extensive thrombosis with significant morbidity and mortality.^154^ Nonetheless, bioactive strategies do not prevent biofouling but instead counteract their downstream consequences. While heparin mitigates thrombosis, other specific bioactive modifications would be simultaneously required to neutralize immune responses, calcification, and infection.

#### 5.2.2 Passive surface modifications

In comparison, passive strategies simply provide a barrier that blocks interactions between the bulk polymer and the blood,^152^ presenting a universal strategy for mitigating a range of biological events by avoiding biofouling altogether. However, long‐term efficacy of passive strategies relies on the robustness of the barrier‐generating mechanism and the stability of the bonds formed between the surface and bulk materials. Hydrophobic materials generate a passive barrier via entrapped air at the surface, facilitated by their nonpolar chemistry and nanoscale surface roughness.^187^, ^188^ However, increased hydrophobicity has been associated with greater immune responses and thrombosis.^125^ Similarly, so‐called omniphobic materials have been proposed, composed of tethered liquid perfluorocarbons, wherein flexible molecular perfluorocarbons are bound to a surface and lubricated with liquid perfluorocarbons.^155^, ^156^ These surfaces can inhibit fibrinogen adsorption and platelet adhesion in vitro and reduce thrombosis in vivo,^155^ while also demonstrating antibacterial properties by preventing biofilm formation on various surfaces.^155^, ^157^ However, long‐term stability studies are required before such modifications can be applied on indwelling devices such as HVRs.

Alternatively, hydrophilic materials associate a layer of water around the material to generate this passive barrier.^152^ Whitesides et al. empirically analyzed the antifouling performance of a vast library of chemistries and identified hydrophilicity as a critical property of antifouling surfaces.^189^ Other so‐called Whitesides' Rules for antifouling surfaces included the presence of hydrogen‐bond acceptors, absence of hydrogen‐bond donors, and electrical neutrality.^189^ A well‐explored hydrophilic modification is poly(ethylene glycol) (PEG), which generates a physical hydration layer by binding water via hydrogen bonding.^158^, ^159^ It has been proposed that the molecular compression of this hydration layer caused by approaching proteins leads to an entropic decrease and steric repulsion.^159^ Despite demonstrating impressive resistance to biofouling,^160^ the hydration layer is not impervious to blood proteins,^162^ and the PEG molecules itself are susceptible to in vivo oxidative degradation.^158^ Furthermore, anti‐PEG antibody generation has been observed, stimulating immune responses and accelerating the clearance of PEG from the body.^158^

An emerging family of hydrophilic materials are zwitterions, which bind water molecules via ionic interactions^152^, ^190^ and generate significantly superior hydration compared to PEG.^191^ Zwitterions are molecules that contain oppositely charged ionic groups but are electrically neutral overall.^192^ Zwitterions bind water molecules in a similar structural state as they would be in bulk solution, thus making displacement by blood proteins thermodynamically unfavorable, generating an energetic barrier in addition to a physical barrier.^162^, ^163^ Three common zwitterions explored are those containing sulfobetaine,^164^, ^165^, ^169^, ^171^, ^175^, ^176^, ^179^, ^180^, ^183^ carboxybetaine,^164^, ^169^, ^174^, ^178^, ^179^, ^193^, ^194^, ^195^ and phosphocholine^168^, ^170^, ^173^, ^179^ moieties. Zwitterionic modifications significantly diminish biofouling, as illustrated by fractional rates of fibrinogen adsorption,^164^, ^165^, ^166^, ^167^, ^168^, ^169^, ^170^, ^171^, ^172^ platelet adhesion^164^, ^168^, ^173^, ^174^, ^175^, ^176^, ^177^ and macroscopic thrombus formation^168^, ^170^, ^171^, ^173^, ^176^, ^178^ relative to unmodified controls. Like other experimental surface modifications, the long‐term stability of zwitterionic grafts has yet to be investigated, although some shorter‐term stability studies have been reported.^165^, ^170^ For example, a phosphocholine‐based layer grafted on to polyvinyl chloride via amine‐functionalization demonstrated no significant changes after 30 days of agitation in phosphate‐buffered saline.^170^ Interestingly, a sulfobetaine‐based layer grafter on to polyurethane via the Fenton reaction only demonstrated 7 days of stable attachment in distilled water,^165^ suggesting the importance of the grafting strategy in achieving stable surface modifications. Thus, the long‐term stability of zwitterionic grafts in the cardiac environment must be further investigated before they may be used in devices such as PPVRs.

## 6 SOMATIC OUTGROWTH

### 6.1 Patient growth and prosthetic‐patient mismatch

In general, clinical outcomes of RVOT reconstruction identify a younger age at implantation as a significant risk factor in early PPVR failure and reoperation.^19^ In addition to the amplified consequences of biofouling in this age group, this observation may be explained by patient outgrowth of the device.^12^, ^14^, ^18^, ^26^, ^27^, ^196^ Clinically, this is described as prosthetic‐patient mismatch (PPM),^197^ which is generally indicated when the ratio of the valve's effective orifice area (EOA, a measure of the cross‐sectional area open to blood flow during systole) to the patient's body surface area decreases below 0.85 cm^2^/m^2^.^198^, ^199^ PPM has a detrimental effect on the hemodynamic profile around the valve, resulting in impaired cardiovascular function and reduced quality of life,^24^ and requiring reoperation to upsize the valve.^27^ The PPM may also exacerbate biological responses to the device, as a progressively non‐physiological flow profile through the HVR may promote thrombosis.^200^, ^201^ Additionally, PPM has been correlated to greater rates of SVD in bioprosthetics.^198^, ^199^ Younger age at implantation has universally been associated with poorer prognoses,^61^, ^62^, ^74^, ^120^, ^202^ which may be partly due to the increased incidence of PPM associated with younger patients.^203^ Indeed, cardiovascular structures grow substantially in the first 20 years of life; importantly to PPVRs, the diameter of the PV annulus typically enlarges from 7 to 24 mm over this period.^204^, ^205^, ^206^ It should be noted that somatic outgrowth has been proposed as a minor concern compared to valve degradation^207^; however, this finding more accentuates the challenge of adverse biological reactions than it does diminish the challenge of somatic outgrowth. Some reports suggest that valve insufficiency may be delayed by oversizing the PPVR implantation,^13^, ^208^, ^209^ which typically involves implanting a valve that is two sizes larger than the patient anatomy (e.g., implanting a valve with a 14 mm diameter for a 10 mm diameter anatomy); however, others refute the long‐term usefulness of this strategy.^210^, ^211^

### 6.2 Growth‐accommodating PPVRs

A growth accommodating PPVR would facilitate remarkable advances in the treatment and management of CHDs by undergoing structural expansion to match its growing environment, while maintaining its hydrodynamic function. Various prototypes have been proposed for this purpose, including biological venous valves, tissue engineered heart valves (TEHVs), and purely synthetic growth‐accommodating heart valves (SGHVs) (Table 3).

**TABLE 3:** In vivo studies for growth accommodating HVR prototypes.

					Diameter increase				
Design	Type	Material	Host species	Expansion method	%	Effect on EOA	Effect on transvalvular pressure	Effect on valvular insufficiency	Other comments
Bovine venous valve^89^	Bioprost hetic	Bovine trileaflet jugular venous valve in a platinum‐iridium stent	Human	Implantation in a slightly compressed state, followed by transcatheter balloon dilation upon patient growth	14	Not reported	Decreased after valve dilation (increased transvalvular pressure was the indicator for dilation)	Insufficiency with a range of severity grades was recorded, but no difference was observed before and after dilation	Off‐label investigation on the growth capacity of the Melody valve
Human venous valve^212^	Bioprost hetic	Cryopreserved femoral human venous vein in a platinum‐iridium stent	Human	Implantation in a slightly compressed state, followed by transcatheter balloon dilation upon patient growth	33	Not reported	Not reported	Mild regurgitation observed in both patients.	Only tested in two patients, and no results beyond 4 months
Trileaflet ECM scaffold^213^	TEHV	Lab‐grown decellularized ECM as a scaffold to guide tissue regeneration	Lamb	Cellular remodeling in response to patient growth	32	Increased with diameter	Remained low (5–10 mmHg) throughout expansion	Remained mild throughout expansion, although gradually increased with diameter	Mechanical properties were modified during remodeling, incomplete remodeling and some calcification was observed. The final valve diameter corresponded to a fully grown sheep
Trileaflet supramolecular polymeric scaffold^214^	TEHV	Supramolecular polymer, designed to resorb while native tissue regenerates	Human	Cellular remodeling in response to patient growth	0	Not reported	Largely remained between 10–20 mmHg	Slight increase from “none or mild” to “mild or moderate” after 1 year	No increase in valve diameter was observed, although this may be attributed to slow human growth rates and a short study period
Bioinspired synthetic bileaflet^110^	SGHV	ePTFE leaflets in a stainless‐steel stent	Lamb	Large coaptation area of bileaflet design facilitates closure as diameter increases. Transcatheter balloon dilation used to expand the device in situ	60	Increased with diameter	Remained low (~10 mmHg) throughout expansion	Regurgitant fraction remained low (5%–8%) throughout expansion	No adverse biological events and no side effects from transcatheter expansion

#### 6.2.1 Venous valves

Due to variable blood flow rates through the venous system, venous valves exhibit the capacity to function over a range of diameters due to greater height to diameter ratios and longer leaflet free edges than semilunar cardiac valves.^215^, ^216^ Trileaflet bovine venous valves, like the Melody valve, have demonstrated slight in situ overdilation; however, the largest reported expansion is only 14%,^89^ which is far from the observed cardiac growth of 100%–200%. Similarly, cryopreserved bileaflet human venous veins in a bare metal stent have reported an in vivo expansion of 33%; however, dilation resulted in mild regurgitation and no results longer than 4 months post‐implant have been reported.^212^ Nonetheless, these biological valves remain susceptible to SVD and other limitations of bioprosthetics, barring their application as a long‐term PPVR.

#### 6.2.2 Tissue engineered heart valves

TEHVs consist of an acellular polymeric or biological valve scaffold that guides cellular integration,^217^ facilitating the regeneration of a tissue heart valve that may theoretically grow with the patient.^213^, ^218^, ^219^, ^220^ For example, a recent TEHV constructed from a lab‐grown decellularized ECM exhibited adequate hydrodynamic performance for over a year in growing lambs.^213^ This material was developed by culturing fibroblasts in fibrin gel in a tubular mold,^221^ resulting in circumferentially‐aligned ECM components and biomimetic anisotropic mechanical properties.^218^, ^222^ Early pre‐clinical characterization demonstrated no calcification,^223^ extensive recellularization,^221^, ^223^, ^224^ and structural growth over time.^225^, ^226^ A valve constructed from this material was implanted in the PV position of 4‐month‐old lambs, where its diameter and EOA gradually enlarged over 1 year as the lambs grew into adulthood.^213^ In fact, leaflet height was maintained over the year and the leaflet free‐edge actually increased in length,^213^ which is a significant observation considering that leaflet contraction has been historically associated with TEHVs.^225^, ^227^, ^228^, ^229^ However, incomplete recellularization was observed and the preprogramed mechanical properties in the recellularized regions were altered in vivo,^213^ with both observations reinforcing earlier findings,^223^, ^224^, ^225^, ^230^ and a gradual increase in valvular insufficiency was observed.^213^ Nonetheless, the valve was able to accommodate the complete growth of the lamb (correlating to a diameter increase of 32%) without progressing past moderate regurgitation,^213^ demonstrating potential for further clinical evaluations. Similarly, a polymeric TEHV has been developed by Xeltis (https://xeltis.com/) using supramolecular polymers,^214^, ^231^, ^232^, ^233^ which were designed to resorb after implantation while simultaneously inducing cellular remodeling to regenerate a biological valve.^231^, ^232^ Through electrospinning, these scaffolds were bestowed with biomimetic anisotropic mechanical properties^228^, ^229^; however, the scale of this anisotropy was diminished during in situ remodeling in pre‐clinical studies.^229^ In a small‐scale clinical trial involving implantation in the pulmonary position of pediatric patients (median age of 5 years), 92% of participants experienced moderate or severe valve regurgitation within 12 months, attributed to unbalanced scaffold degradation and cellular repopulation processes.^233^ By thickening the scaffold leaflets, a second small‐scale pediatric clinical trial only saw 17% of patients exceed mild regurgitation after 12 months,^214^ promoting current recruitment for a larger‐scale trial (NCT03022708). No significant increase in valve diameter was observed over this period^233^; however, this is likely due to slow human grow rates and a relatively short study period. To this end, the planned clinical trial will be pivotal in evaluating the utility of the Xeltis valve as a growth accommodating PPVR.

Critically, the clinical translation of TEHVs is largely limited by a lack of cellular control within the implanted scaffold. A common observation is the contraction of neo‐tissue in the recellularized leaflets, resulting in leaflet shortening and valve insufficiency.^225^, ^227^, ^228^, ^229^ This is a particular trepidation for pediatric applications as valve insufficiency would likely be exacerbated by vascular growth. Another concern is the significantly heterogeneous remodeling rates both within and between valves,^232^, ^234^ which bars certainty regarding the composition and quality of the remodeled valve. Consequently, recent work has explored strategies to better predict and control the quality of the remodeled valve, whether through optimizing the design and fabrication process,^39^, ^235^ or better understanding the underlying cellular processes.^232^, ^236^ For example, computational simulations have been employed to predict leaflet shortening in TEHVs, allowing for the design of a geometry that anticipated these structural changes.^39^ The projected leaflet shortening was observed within 6 months of implantation in sheep, and regurgitation mostly remained under 20% for the length of the 52‐week study.^39^ Experimental evidence of remodeling was similar to the computational predictions,^39^ and this approach facilitated less macrophage infiltration, less cellular expression of contractile proteins, and faster endothelialization rates than non‐modeled TEHVs.^237^ Additionally, M2 macrophages^232^, ^236^ and FBGCs^232^ may be critical cellular participants in balancing scaffold resorption and tissue regeneration, suggesting that managing these cell types might facilitate greater control over the remodeling process. Such work is integral to developing a comprehensive understanding of the remodeling process; however, the current lack of control over cellular remodeling imposes safety challenges that hinder the clinical translation of TEHVs.^238^

#### 6.2.3 Synthetic growth‐accommodating heart valves

SGHVs describe purely synthetic polymeric valves that rely on innovative designs to facilitate expansion in lieu of organic remodeling. While some theoretical prototypes have been reported,^239^ to the best of the authors' knowledge only one SGHVs has been fabricated; a bileaflet valve consisting of ePTFE leaflets on a bare stainless‐steel stent, with an expansile capacity of 180%.^110^ This design was based on computational simulations that demonstrated that bileaflet venous valves could expand up to three times as much as trileaflet semilunar valves while preserving hydrodynamic function.^216^ As the diameter of this prototype increased in vitro, the EOA and flow rate also increased, while preserving adequate transvalvular pressure drops and regurgitant fractions. A 10‐week in vivo investigation in the PV position of growing lambs demonstrated valve expansion from 12.5 to 20 mm (via transcatheter balloon dilation) as lamb weight increased from 20 to 60 kg. Biomimetic hemodynamic profiles were preserved throughout the study, and no adverse biological events occurred.^110^ Demonstrating promising progress toward a durable PPVR, longer‐term in vivo studies or small population clinical trials could offer further insight regarding its suitability for clinical translation.

Further development of SGHVs may benefit from inspiration from other growth‐accommodating medical devices; the challenge of growth affects almost all pediatric prosthetics, such as vascular grafts, myocardial patches and tracheal grafts.^220^ For example, structural supports with growth capacity have been developed with a biostable fiber mesh encasing a biodegradable core, allowing for the mesh to reconfigure and extend as the interior core degrades with time.^240^ However, as somatic growth is highly heterogeneous between individuals,^204^, ^205^, ^206^ incorporating time‐dependent biodegradable elements may result in hazardous imbalances between the structural changes of the device and the patient tissue. Alternatively, an expandable vascular graft crafted from a completely biostable ePTFE with directional extensile properties (Stretch Gore‐Tex™) recently demonstrated spontaneous expansion in growing lambs, increasing diameter by 37% as the animals reached adulthood.^241^ While the introduction of leaflets presents more complex structural challenges, these expandable prototypes demonstrate progress toward long‐term surgical therapies for CHDs. With increased interest in this challenge, functional growth‐accommodating PPVRs may become clinically feasible, whether through the advancements in biological modeling required for TEHVs, or the integration of progressive manufacturing strategies, biocompatible polymers, and innovative designs required for SGHVs.

## 7 CONCLUSIONS AND FUTURE PERSPECTIVES

A next‐generation PPVR with reduced reoperation requirements would help to alleviate the persistent physical, financial, and mental burdens of many CHDs, presenting an urgent challenge that necessitates strong collaborations between clinicians and engineers. Specifically, these devices require the development of suitable antifouling biomaterials, and designs that accommodate patient growth. In response to the former challenge, the surface of mechanically suitable biomaterials may be modified with materials with outstanding antifouling capabilities. Passive hydrophilic modifications such as zwitterions may offer an enduring strategy, and the evolving mechanistic understanding of zwitterionic chemistry facilitates increasingly effective molecular designs.^242^ While a range of grafting techniques are known to facilitate surface modifications, such as wet‐chemical approaches exploiting dopamine chemistry^170^, ^180^, ^243^ or dry‐chemical strategies such as plasma treatment,^244^, ^245^, ^246^ extended stability studies are required to validate the resilience of these strategies in the dynamic cardiac environment. Simultaneously, PPVRs need to grow with the patient, demanding either advancements in cellular remodeling expertise to improve TEHV quality, or the conception of original SGHV prototypes. In this respect, computational modeling would provide a critical tool, based on its integral role in the development of various recent HVR prototypes.^39^, ^110^, ^216^, ^239^, ^247^ In this review alone, simulations have accurately predicted structural changes due to cellular contraction,^39^ compared the expansile capability of various biological valves,^216^ and analyzed the mechanical performance of a bileaflet SGHV.^110^ In silico studies could readily analyze the functional performance of an expanding prototype without committing excess time to manufacture, thus streamlining the iterative development of growth‐accommodating PPVRs. As this technology evolves, simulation components may allow the application of artificial intelligence and digital twins,^248^, ^249^ wherein the child's growth could be predicted and PPVR expansion modeled with unprecedented patient‐specificity. In addition, an emerging range of so‐called “meta‐biomaterials” offer unique and non‐intuitive mechanical properties^250^ that might permit PPVR expansion. For example, auxetic materials can expand simultaneously along two perpendicular axes due to carefully engineered micro‐ or nanoscale architectures, whereas normal materials tend to shrink in the axis perpendicular to that being stretched.^251^, ^252^ Such distinctive material properties could potentially facilitate the three‐dimensional reconfiguration of an expanding PPVR.

Ultimately, polymeric valves offer the greatest strategy for PPVR development. The unique processibility of polymers has permitted the fabrication of various polymeric valve prototypes with advanced manufacturing techniques, such as 3D printing,^253^, ^254^ injection molding,^113^ electrospinning^228^ and melt electrowriting.^255^ Accordingly, these strategies would readily allow the fabrication of growth accommodating PPVRs from antifouling biomaterials. Importantly, a significant barrier facing next‐generation polymeric PPVRs is the lack of precedent for polymeric valves in common clinical use. Beyond a few small‐population trials,^109^, ^117^, ^214^ polymeric valves have yet to definitively demonstrate their clinical efficacy in the adult population, let alone the pediatric population. Additionally, polymeric PPVRs should be designed to allow transcatheter deployment to match the current state of the art,^54^ while maintaining surgical options for younger patients. Despite these challenges, progress toward next‐generation PPVRs should be pursued to alleviate some of the burdens associated with CHDs. Indeed, beyond reducing physical and financial burdens associated with surgery and management, such a device would relieve the psychological uncertainty over the longevity of each treatment, facilitating a better quality of life for both the patient and the parents. Additionally, many other valvular heart diseases burden the pediatric and adult populations^10^, ^256^ and progress toward satisfactory PPVRs could provide a platform to launch innovations in other HVRs. Furthermore, the successful development of antifouling biomaterials could find application in extending the lifespan of various cardiovascular devices such as vascular catheters, stents, ventricular assist devices or extracorporeal membrane oxygenation units,^63^ while an improved understanding of growth accommodating designs could benefit many other pediatric implants.^196^, ^220^ Thus, next‐generation PPVRs would meet an urgent clinical challenge in the treatment of CHDs, while establishing precedent for the invention of better pediatric and cardiovascular medical technologies.

## AUTHOR CONTRIBUTIONS

**Matthew Alexander Crago:** Conceptualization (lead); data curation (lead); investigation (lead); visualization (lead); writing – original draft (lead). **David Scott Winlaw:** Funding acquisition (equal); supervision (supporting); validation (lead); writing – review and editing (lead). **Syamak Farajikhah:** Writing – review and editing (supporting). **Fariba Dehghani:** Funding acquisition (equal); resources (lead); writing – review and editing (supporting). **Sina Naficy:** Conceptualization (supporting); funding acquisition (equal); project administration (lead); resources (supporting); supervision (lead); writing – review and editing (equal).

### PEER REVIEW

The peer review history for this article is available at https://publons.com/publon/10.1002/btm2.10501.

## Data Availability

Data available on request from the authors.
